# Study of the direct medical economic burden of patients with healthcare-associated infection based on DRGs matching

**DOI:** 10.1186/s12913-026-14732-7

**Published:** 2026-05-16

**Authors:** Yujing Zhang, Zhenhua Duan, Zhenhan Mo, Jianjun Xu, Han Zhang

**Affiliations:** 1 Department of Hospital Infection and Control, Chengdu First People’s Hospital, 18, Wanxiang North Road, Chengdu, Sichuan Province 610041 China; 2 Department of AIDS & STD Control and Prevention, Chengdu Center for Disease Control and Prevention (Chengdu Institute of Health Supervision), 4, Longxiang Road, Chengdu, Sichuan Province 610041 China

**Keywords:** Diagnosis related groups, Healthcare-associated infection, Direct medical economic burden

## Abstract

**Background:**

To discuss the direct economic burden of Healthcare-associated Infection (HAI) in a hospital in Chengdu.

**Methods:**

All patients enrolled in the Diagnosis Related Groups (DRGs) comprehensive evaluation and management system between January 1 and December 31, 2023, were categorized into two groups: those with a Healthcare-associated Infection (HAI) and those without. A 1:1 case matching was performed based on DRG classification, sex, and age (± 5 years). Differences in direct medical costs and length of stay (LOS) between the two groups were compared, followed by a subgroup analysis of infection-related economic burden across diagnostic categories.

**Results:**

The HAI incidence rate was 0.92%, and the HAI rate per 1,000 patient days was 1.23‰ in 2023. 726 pairs of HAI cases and non-HAI cases were successfully matched. The direct cost of the HAI group was CNY 16,251.63(13244.10 ~ 19259.16) higher than the non-HAI group (1.91 times higher, *P* < 0.001). The length of hospital stay in the HAI group was 10(8.58 ~ 11.42) days longer (1.71 times higher, *P* < 0.001), with significant cost increases in treatment fees and Western medicine (*P* < 0.001). Among all Major Diagnostic Categories (MDCs), the infection and parasitic diseases group incurred the largest economic burden. For HAI patients with sepsis or severe sepsis requiring ≥ 96 h of mechanical ventilation, the total cost was CNY 80,162.31(27439.11 ~ 132885.51) higher than non-HAI patients (*P* = 0.003), and hospitalization duration increased by 17.5(5.99 ~ 29.01) days (*P* = 0.003).

**Conclusions:**

In conclusion, implementing scientifically classified DRGs in HAI management provides a strategic framework for advancing precision-oriented healthcare administration.

## Introduction

To enable more scientific allocation of medical resources and standardization of healthcare practices, the National Healthcare Security Administration of China has drawn on international experience in recent years to deepen the reform of medical insurance payment methods [1]. In 2021, the General Office of the State Council of China issued the “14th Five-Year” National Medical Security Plan, emphasizing the continuous advancement of medical insurance payment reforms. It proposed the nationwide implementation of a diversified composite payment system, primarily based on diagnosis-related groups (DRGs) payment, to guide rational clinical practices and enhance the efficiency of healthcare fund utilization [2]. In the same year, the National Healthcare Security Administration of China released the Three-Year Action Plan for DRG/Diagnosis-Intervention Pocket (DIP) Payment Reform, highlighting a shift from the traditional “fee-for-service” model to a “diagnosis-related group payment” model as the cornerstone of medical insurance reform [1].

DRGs serve as a critical tool for reforming medical insurance payments and guiding the rational allocation of healthcare resources. According to the Technical Specifications for Chinese Healthcare Security Diagnosis-Related Group (CHS-DRG) Classification and Payment, DRG is a case-mix classification system. Cases are first categorized into Major Diagnostic Categories (MDCs) based on distinct disease types. Within the same MDC, cases are further differentiated into core DRG groups according to treatment methods. Finally, individual patient characteristics, such as age, complications, comorbidities, and birth weight, refine the grouping to establish specific DRG sets [3]. DRGs are instrumental in evaluating healthcare quality, efficiency, and medical insurance payments. Under this new payment model, medical institutions bear the additional economic burden caused by Healthcare-Associated Infections (HAIs), as the fixed DRG-based payment does not account for excess costs from HAIs.

HAIs are among the most common adverse events in healthcare delivery, imposing a significant burden on health systems [4]. The DRG payment reform will inevitably drive hospitals to adopt more refined management practices. Understanding the disease spectrum distribution, high-impact conditions, resource consumption patterns, and time utilization within medical institutions, as well as quantifying the economic losses attributable to HAIs, is essential for informing hospital resource allocation and infection prevention strategies.

## Methods

### Study population and study design

**Fig. 1:**
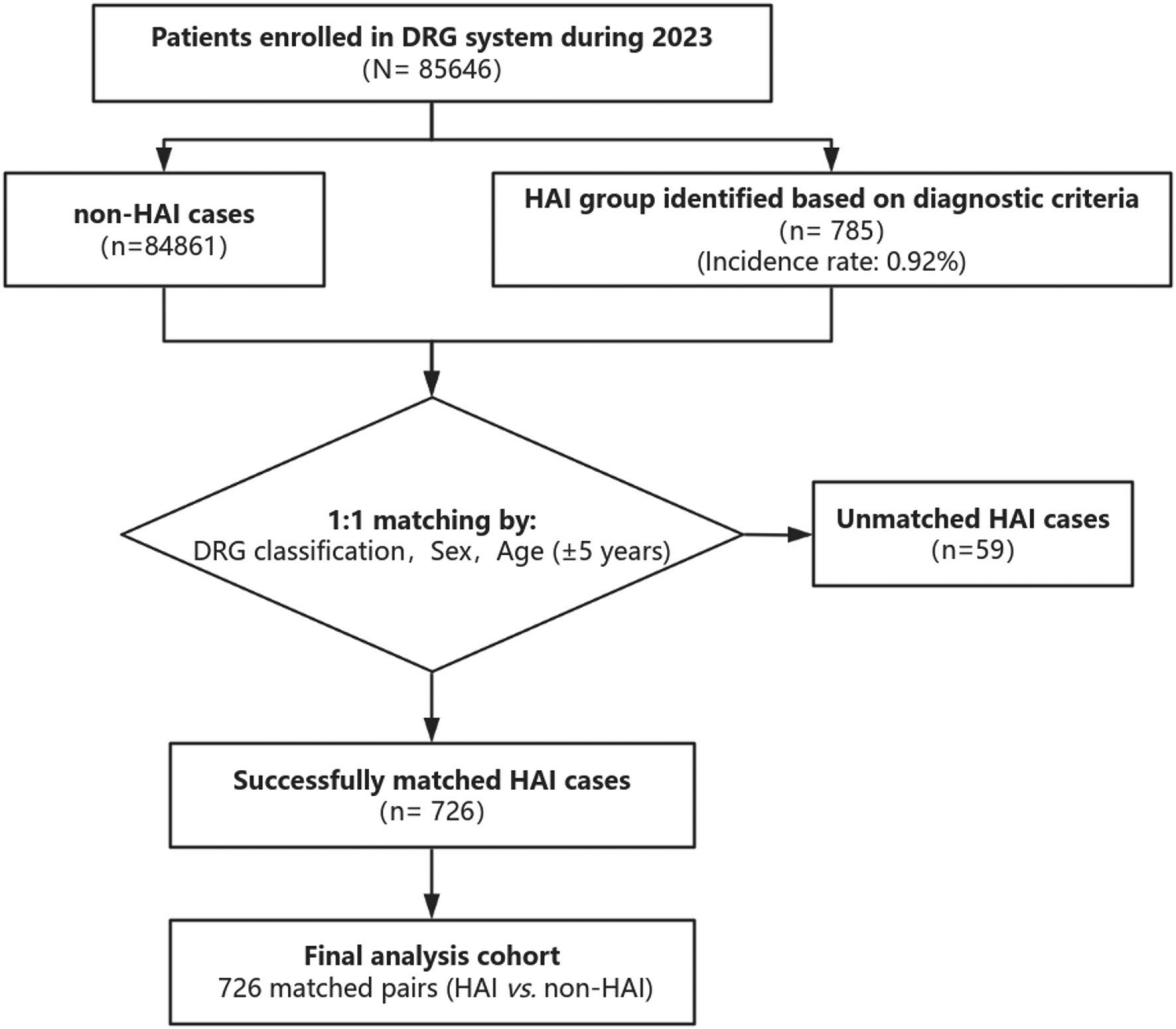
Flowchart of patient selection and 1:1 matching process

This study retrospectively analyzed all discharged cases (January 1 to December 31, 2023) from a tertiary hospital in Chengdu enrolled in the hospital DRG Comprehensive Evaluation and Management System. The Healthcare-Associated Infection Diagnostic Criteria used in this study were issued by the Health and Family Planning Commission of China in 2001 and the timing threshold for HAI classification is > 48 h post-admission [5]. Based on the HAI Diagnostic Criteria, subjects were stratified into HAI (785 cases) and non-HAI (84,861 cases) groups. Data on MDCs and DRG classifications were extracted from the DRG system (see Fig. 1). At the same time, infection timing/diagnosis details were retrieved from the infection surveillance system, and direct medical economic burden metrics (hospitalization duration, total costs, treatment expenses) were sourced from the hospital management information system. HAI patients spanned 196 DRG groups; a 1:1 case matching (gender, age ± 5 years, DRG group) achieved 726 matched pairs (92.48% success rate), reducing post-match HAI coverage to 172 DRG groups.

### Outcome measures

The direct medical economic burden refers to the economic resources consumed in purchasing healthcare services, encompassing outpatient fees, hospitalization fees, medication costs, and other disease prevention/treatment expenses. Differences in direct economic burden and hospitalization duration between matched HAI and non-HAI cases under the CHS-DRG payment framework were analyzed. Subgroup analyses were further conducted for MDC categories and DRG groups with high HAI incidence within the hospital.

### Statistical analysis

R version 4.3.3 was used for data analysis. Categorical data were presented as frequencies, with intergroup comparisons using the χ² test or Fisher’s exact probability test. Median (M) and interquartile range (IQR) were reported for skewed continuous data. Differences between groups were assessed using the Mann-Whitney U test. A significance level of *P* < 0.05 was applied.

## Results

### Patient characteristics

From January 1 to December 31, 2023, 85,646 hospitalized patients were enrolled in the DRG system, with a cumulative hospitalization duration of 639,932 days. Among them, 785 cases were classified into the HAI group and 84,861 into the non-HAI group. The HAI incidence rate was 0.92%, and the HAI rate per 1,000 patient days was 1.23‰. Before case matching, significant differences were observed in gender and age distribution between the two groups (*P* < 0.001, see Table 1). After matching, 726 pairs of HAI and non-HAI cases were formed, covering 172 DRG groups across 23 MDCs, and no significant differences were found in gender, age, or DRG group distribution (all *P* > 0.05, see Table 1).

**Table 1:** Sex and age distribution of HAI and non-HAI patients before and after matching

Characteristics	Before matching				After matching			
	HAI	non-HAI	Z	*P*	HAI	non-HAI	Z	*P*
Gender								
Male	434	41,585	14.496	0.001	404	404	0	1
Female	351	43,276			321	321		
Age [M, IQR]								
	68(76)	56(69)	-14.833	< 0.001	68(23)	68(21)	-0.38	0.71

### Direct medical economic burden of HAIs

The median hospitalization duration for HAI patients was 24.00 days, 1.71 times longer than that of non-HAI patients. The median total hospitalization cost for HAI patients was CNY 34,056.08, 1.91 times higher than that of non-HAI patients. Treatment fees (CNY 2,992.24) and Western medicine expenses (CNY 2,930.46) were the largest cost increases. Except for traditional Chinese medicine costs, all other expense categories—bed fees, nursing fees, Western medicine, laboratory tests, surgery, treatment, and examination fees—were significantly higher in the HAI group compared to the non-HAI group (all *P* < 0.001, see Table 2).

**Table 2:** Direct cost and hospital stay between HAI group and non-HAI group (CNY)

Item	HAI(*n* = 726)		non-HAI(*n* = 726)		Difference	Statistic	
	M	IQR	M	IQR	d 95% CI	Z	*P*
Length of Stay	24.00	39.00	14.00	23.00	10.00 (8.58 ~ 11.42)	-13.826	<0.001
Total Cost	34,056.08	49,663.60	17,804.45	28,177.43	16,251.63 (13244.10 ~ 19259.16)	-10.596	<0.001
Ward Charge	1,341.00	1,524.50	729.00	845.50	612.00 (527.49 ~ 696.51)	-14.197	<0.001
Treatment Fee	6,276.56	1,1631.63	3,284.32	5,929.55	2,992.24 (2420.59 ~ 3563.89)	-10.258	<0.001
Diagnostic Imaging Fee	2,079.50	2,862.25	1,425.00	1,868.00	654.00 (466.69 ~ 842.31)	-6.826	<0.001
Nursing Care Fee	830.00	1,317.00	442.00	595.50	388.00 (325.38 ~ 450.62)	-12.145	<0.001
Consultation Fee	428.00	432.00	258.00	256.00	170.00 ()146.30 ~ 193.70	-14.044	<0.001
Laboratory Test Fee	3,688.00	6,491.25	2,105.00	3,650.50	1,583.00 (1322.45 ~ 1843.55)	-11.895	<0.001
Western Medication Cost	6,797.58	11,142.96	3,867.12	7,456.31	2,930.46 (2246.29 ~ 3614.63)	-8.387	<0.001
Medical Supplies Cost	1,084.60	5,063.29	517.11	2,868.55	567.49 (406.48 ~ 728.50)	-6.913	<0.001
Chinese Patent Medicine Cost	680.04	1,581.08	473.60	1,138.28	206.44 (-964.11 ~ 1376.99)	-0.345	0.73

Note: Abbreviations, M, median; IQR, interquartile range

### HAI burden in MDC subgroups

Matched HAI cases covered 172 DRG groups across 23 MDCs. As Table 3 shows subgroup analysis focused on MDC groups with ≥ 20 HAI cases. The largest HAI subgroup was “Myeloproliferative Diseases and Dysfunction, Poorly Differentiated Neoplasms” (138 cases, 19.01% of total HAI cases), with an HAI incidence of 1.98%. In this subgroup, total hospitalization costs for HAI patients were CNY 14,651.75 higher (*P* < 0.001), and hospitalization duration was 11.50 days longer (*P* < 0.001) compared to non-HAI cases. The most significant differences in total costs (CNY 37,543.20, *P* < 0.001) and hospitalization duration (22.00 days, *P* < 0.001) were observed in the “Infectious and Parasitic Diseases (Systemic or Unspecified)” MDC. Except for “Circulatory System Diseases and Dysfunctions,” all other MDCs showed statistically significant differences in total costs and hospitalization duration between HAI and non-HAI groups (*P* < 0.05).

**Table 3:** Direct cost and hospital stay between HAI group and non-HAI group in different MDC group

Major Diagnostic Categories	Number of HAI Cases	Total Medical Cost (CNY)					Median Length of Stay (days)				
		HAI	non-HAI	Difference 95% CI	Z	*P*	HAI	non-HAI	Difference 95% CI	Z/t	*P*
Myeloproliferative Diseases and Disorders and Poorly Differentiated Neoplasms	138	30,806.19	16,154.44	14,651.75 (8581.11 ~ 20722.39)	-4.725	< 0.001	26.00	14.50	11.50 (7.92 ~ 15.08)	-6.313	< 0.001
Diseases and Disorders of the Nervous System	135	49,989.93	21,158.03	28,831.90 (16694.11 ~ 40969.69)	-4.658	< 0.001	31.00	16.00	15.00 (9.99 ~ 20.01)	-5.870	< 0.001
Diseases and Disorders of the Respiratory System	96	30,274.77	16,908.73	13,366.04 (7521.11 ~ 19210.97)	-4.483	< 0.001	20.00	16.50	3.50 (1.52 ~ 5.48)	-3.467	< 0.001
Diseases and Disorders of the Digestive System	68	31,932.69	16,524.70	15,407.99 (6994.11 ~ 23821.87)	-3.599	< 0.001	23.00	12.50	10.50 (6.46 ~ 14.54)	-5.092	< 0.001
Diseases and Disorders of the Musculoskeletal System	46	26,920.48	18,392.64	8,527.84 (166.11 ~ 16889.57)	-1.999	0.046	21.00	12.00	9.00 (4.62 ~ 13.38)	-4.025	< 0.001
Infectious and Parasitic Diseases (Systemic or Unspecified Sites)	40	69,914.79	32,371.59	37,543.20 (15671.11 ~ 59415.29)	-3.368	< 0.001	34.00	12.00	22.00 (13.75 ~ 30.25)	-5.224	< 0.001
Diseases and Disorders of the Kidney and Urinary System	34	30,452.64	17,741.42	12,711.23 (2021.11 ~ 23401.35)	-2.330	0.020	29.26	18.06	11.20 (4.07 ~ 18.33)	-3.079	0.003
Diseases and Disorders of the Circulatory System	30	21,262.62	18,522.92	2,739.71 (-5524.89 ~ 11004.31)	-0.651	0.515	17.5	14.13	3.37 (-1.31 ~ 8.05)	-1.412	0.163
Diseases and Disorders of the Liver, Biliary Tract, and Pancreas	29	28,572.15	12,796.39	15,775.76 (8151.11 ~ 23400.41)	-4.051	< 0.001	25.00	13.00	12.00 (5.95 ~ 18.05)	-3.893	< 0.001
Diseases and Disorders of the Blood, Blood-Forming Organs, and Immunity	24	17,632.58	10,238.30	7,394.29 (2704.11 ~ 12084.47)	-3.093	0.002	23.92	12.17	11.75 (4.99 ~ 18.51)	-3.406	0.002

Note: Abbreviations, M, median; IQR, interquartile range

All comparisons were performed using the Mann–Whitney U test

### Subgroup analysis of high-burden DRGs

HAI cases were distributed across four DRG groups for the MDC “Infectious and Parasitic Diseases (Systemic or Unspecified),” which exhibited the highest cost and duration disparities **(**Table 4**)**. Two DRGs-“Sepsis or Severe Sepsis with 96 Hours of Mechanical Ventilation” and “Sepsis or Severe Sepsis without 96 Hours of Mechanical Ventilation but with Major Complication & Comorbidity (MCC)”-accounted for 80% of HAI cases in this MDC.

**Table 4:** Direct cost and hospital stay between HAI group and non-HAI group in different DRG group

Item	HAI(*n* = 726)		non-HAI(*n* = 726)		Difference 95% CI	Statistic	
	M	IQR	M	IQR		Z	*P*
**Sepsis/Severe Sepsis with Mechanical Ventilation ≥ 96 h**							
Length of Stay	32	33	14.5	15	17.5 (5.99 ~ 29.01)	-2.982	0.003
Total Cost	144,459.56	127,633.38	64,297.25	42,484.98	80,162.31 (27439.11 ~ 132885.51)	-2.977	0.003
Ward Charge	2,640.00	2,876.00	1,125.50	1,214.25	1,514.50 (577.81 ~ 2451.19)	-3.166	0.002
Treatment Fee	36,178.24	24,887.63	15,842.68	9,693.86	20,335.56 (8311.11 ~ 32360.01)	-3.317	< 0.001
Diagnostic Imaging Fee	3,815.00	1,856.50	1,478.00	1,848.50	2,337.00 (799.71 ~ 3874.29)	-2.977	0.003
Nursing Care Fee	5,260.66	10,565.83	3,013.76	2,803.60	2,246.90 (266.71 ~ 4227.09)	-2.224	0.026
Consultation Fee	623.00	342.00	414.00	377.50	209.00 (44.51 ~ 373.49)	-2.488	0.013
Laboratory Test Fee	19,906.75	16,105.63	11,050.75	7,125.00	8,856.00 (2803.79 ~ 14908.21)	-2.864	0.004
Western Medication Cost	37,426.68	59,123.94	20,677.99	21,047.32	16,748.70 (-1019.91 ~ 34517.31)	-1.847	0.065
Medical Supplies Cost	12,777.30	16,625.92	3,469.26	5,457.36	9,308.04 (2491.11 ~ 16124.97)	-2.676	0.007
**Sepsis/Severe Sepsis without Mechanical Ventilation ≥ 96 h (with MCC)**							
Length of Stay	32	56	7	9	25 (11.41 ~ 38.59)	-3.604	< 0.001
Total Cost	52,321.08	99,250.71	11,843.52	16,238.47	40,477.55 (7591.11 ~ 73363.99)	-2.412	0.016
Ward Charge	1,999.00	3,379.50	411.5	332.50	1,587.50 (705.11 ~ 2469.89)	-3.526	< 0.001
Treatment Fee	11,904.14	39,919.015	2,437.5	4,951.21	9,466.64 (2124.11 ~ 16809.17)	-2.525	0.012
Diagnostic Imaging Fee	3,169.00	2,746.80	790.00	740.00	2,379.00 (1076.49 ~ 3681.51)	-3.579	< 0.001
Nursing Care Fee	2,382.24	45,074.97	373.50	721.40	2,008.74 (616.04 ~ 3401.44)	-2.827	0.005
Consultation Fee	838.00	922.50	173.00	226.00	665.00 (305.11 ~ 1024.89)	-3.618	< 0.001
Laboratory Test Fee	9,886.50	9,637.50	3,499.50	3,366.63	6,387.00 (1352.49 ~ 11421.51)	-2.487	0.013
Western Medication Cost	11,465.58	1,980.88	3,796.34	6,981.37	7,669.245 (0.01 ~ 15338.49)	-1.960	0.05
Medical Supplies Cost	1,469.29	2,480.63	447.36	932.27	1,021.93 (229.11 ~ 1814.75)	-2.525	0.012

Note: Abbreviations, M, median; IQR, interquartile range

Sepsis/Severe Sepsis with Mechanical Ventilation (96 h):

HAI patients incurred total hospitalization costs of CNY 144,459.56, CNY 80,162.31 higher than non-HAI cases (*P* = 0.003). Their hospitalization was 32.0 days, 17.5 days longer than non-HAI patients (*P* = 0.003). Significant differences were observed in bed fees, treatment fees, examination fees, nursing fees, laboratory fees, and material costs (all *P* < 0.05), except for Western medicine expenses (*P* = 0.065). Treatment fees for HAI patients were 2.28 times higher (CNY 20,335.56), and Western medicine costs 1.81 times higher (CNY 16,748.70) than non-HAI patients.

Sepsis/Severe Sepsis without Mechanical Ventilation (96 h) with MCC:

The total cost difference between HAI and non-HAI cases was CNY 40,477.55, with the most significant disparity in treatment fees (CNY 9,466.64). All cost categories except Western medicine showed significant differences (all *P* < 0.05).

## Discussion

The reform of medical insurance payment methods and macro-regulation aims to shift regulatory focus from cost containment to dual control of healthcare costs and quality. Through DRG/DIP payment reforms, establishing efficient payment management and incentive mechanisms for medical institutions is the starting point and core objective of these reforms [6]. Under the DRG-based fixed payment model, insurance does not cover additional costs caused by HAIs, which incentivizes healthcare providers to improve infection control practices. However, this also raises higher demands for infection prevention infrastructure and specialized personnel. Identifying risks, reducing HAI incidence, alleviating disease burden, and enhancing operational efficiency remain critical priorities [7].

To better assess the disease burden of HAIs while balancing confounding factors between HAI and non-HAI cases, prior studies matched patients based on gender, age, department, or propensity scores [8, 9]. However, HAI patients often present with more severe conditions and prolonged exposure risks, introducing bias. As a scientific grouping and evaluation tool, DRGs refine case classifications based on diagnoses, procedures, comorbidities, and demographic features. Patients within the same DRG group are assumed to have comparable hospitalization durations without confounding factors. Thus, our DRG-based 1:1 matching approach offers greater precision and scientific rigor than traditional methods limited by partial confounder control. This ensures enhanced comparability in diagnoses and demographics, yielding results closer to real-world impacts.

HAIs impose a significant economic burden, directly increasing patient costs and conflicting with healthcare cost-control and quality improvement goals. A study in the EU/EEA estimated the cumulative burden of six HAIs at 501 disability-adjusted life years (DALYs) per 100,000 population (2011–2012), exceeding the total burden of 32 other infectious diseases (including influenza and tuberculosis) reported by the European Centre for Disease Prevention and Control (260 DALYs/100,000 population, 2009–2013) [10]. Studies from various countries have shown that each hospital-acquired infection (HAI) incurs an average cost of $1,000 to $123,000, imposing a substantial economic burden on healthcare systems [11]. For high-risk conditions, HAIs increase costs and prolong hospitalization [12]. The average hospitalization cost for patients infected with multidrug-resistant bacteria is higher than that of non-infected patients [13]. A study conducted at one of the largest tertiary hospitals in Sichuan, China, utilized multivariate analysis to identify confounders affecting hospitalization costs, followed by propensity score matching. The results revealed that hospitalization costs for HAI patients were EUR 2,198.19 higher (1.6-fold increase) compared to non-HAI patients [14], closely aligning with our findings of a CNY 16,251 (1.71-fold) cost increase.

In this study, HAI-induced cost increments varied across MDCs, ranging from CNY 2,700 to over CNY 37,000, with the most significant burdens observed in “Infectious and Parasitic Diseases” and “Nervous System Diseases and Dysfunctions.” Further DRG subgroup analysis revealed the most pronounced disparities in “Sepsis/Severe Sepsis with 96 Hours of Mechanical Ventilation,” where HAI patients faced a CNY 80,162.31 cost increase and 17.5-day hospitalization prolongation. Such high-burden DRG groups warrant prioritized surveillance and targeted infection control measures to mitigate economic and clinical impacts.

Treatment costs constituted the primary driver of additional HAI-related expenses, reflecting increased medical interventions. Medication expenditures, particularly antibiotics, represented the second-largest burden. A meta-analysis by Lv et al. (2009–2019) found medication costs accounting for CNY 9,438 (38%) of the total CNY 24,881 HAI cost difference [15]. Similarly, in India, antibiotics comprised half of HAI-related medication expenses [16]. Furthermore, adjusted discounted costs for HAI patients rose from $3,703.82 in 2016 to $5235.90 in 2022 (a 1.41-fold increase), with annual cost growth rates (~ 7%) outpacing non-HAI cases [17]. Aging populations, invasive procedures, and complex surgeries will likely exacerbate infection risks and economic burdens. Underreporting of HAIs further suggests underestimated true burdens.

An interesting finding in our subgroup analysis was that Western medication costs did not differ significantly between HAI and non-HAI patients in the subgroup requiring prolonged mechanical ventilation. This may be explained by the highly standardized and protocol-driven nature of intensive care in critically ill patients, where pharmacological treatments tend to be similar in type and intensity across individuals, resulting in reduced variability.In addition, previous studies have demonstrated that mechanical ventilation and intensive care interventions are major drivers of ICU costs, often outweighing the contribution of pharmacological treatment alone. Ventilated patients incur substantially higher daily costs, largely due to life-support measures and resource-intensive care rather than medication use [18]. Furthermore, micro-costing analyses have shown that ICU costs are composed of multiple components, including staffing, equipment, and monitoring, with considerable variability across patients. In such settings, non-pharmacological factors often dominate the cost structure, while medication costs contribute a smaller and less variable proportion [19]. Therefore, the lack of statistical significance in Western medication costs in this subgroup does not indicate an absence of treatment differences, but rather reflects the high severity, treatment standardization, and cost structure characteristics of critically ill patients.

Beyond direct costs, HAIs contribute to indirect losses (e.g., lost productivity) and systemic healthcare strain. While many HAIs are preventable, improving compliance with infection control protocols remains resource-intensive [20]. Thus, evidence-based prioritization of HAI burden assessments is essential to optimize resource allocation.

Based on our findings, high-burden DRGs—particularly infectious and parasitic diseases, respiratory system disorders, and sepsis requiring mechanical ventilation—should be prioritized for infection prevention and control (IPC) efforts. Targeted interventions, including enhanced surveillance and antimicrobial stewardship, are warranted in these high-risk populations.

Several limitations should be acknowledged. First, this study was conducted at a single tertiary hospital, which may limit the generalizability of our findings to other healthcare settings with varying patient populations, practice patterns, and reimbursement systems. Second, although we applied a rigorous DRG-based matching approach, 59 HAI cases (7.5%) could not be matched to suitable controls due to the lack of comparable non-HAI patients within the predefined criteria, potentially introducing selection bias. Third, our analysis focused on direct medical costs only; indirect costs and clinical outcomes (e.g., mortality, readmission) were not assessed, which precludes a comprehensive assessment of the total burden attributable to HAIs.

## Conclusion

The utilization of DRGs in the context of hospital-acquired infections offers a direction for precision management. Integrating DRGs into HAI management offers a strategic framework for precision-oriented governance and sustainable healthcare delivery.

## Data Availability

The anonymized aggregate data that support the findings of this study are available from the corresponding author upon reasonable request. Individual-level patient data are not publicly available due to privacy and ethical restrictions.

## Abbreviations

HAI: Healthcare-associated Infection
LOS: Length of stay
MDCs: Major Diagnostic Categories
DRGs: Diagnosis-related groups
DIP: Diagnosis-Intervention Pocket
CHS-DRG: Chinese Healthcare Security Diagnosis-Related Group
M: Median
IQR: Interquartile range
MCC: Major Complication & Comorbidity
IPC: Infection prevention and control
